# Supplementary material for: Entropy and interfacial energy driven self-healable polymers
Source: Nat Commun. 2020 Feb 25;11:1028. doi: 10.1038/s41467-020-14911-y (PMC7042321; doi:10.1038/s41467-020-14911-y)
Supplement: Supplementary file 1 — Supplementary Information [file 41467_2020_14911_MOESM1_ESM.pdf]

Supplementary Materials

Entropy and Interfacial Energy Driven Self-Healable Polymers

Chris C. Hornat and Marek W. Urban*

Department of Materials Science and Engineering,
Center for Optical Materials Science and Engineering Technologies (COMSET)

Clemson University, Clemson, SC 29634 USA

* - corresponding author: mareku@clemson.edu

Supplementary Discussion

Polymerization Reaction

Polymerization reaction leading to TPU synthesis is shown in Supplementary Fig.1. The polymer was drawn to achieve approximately 140 μm diameter fibers. Further details regarding their preparation are provided in the Materials and Methods section.

Dynamic Mechanical Analysis (DMA)

Supplementary Fig. 2 shows that during DMA 72 kDa TPU exhibits a VLT[1] around 42 ^{0}C, and the corresponding stored entropic energy density (ΔS_S = 30.7 kJ/m^3), maximum stored strain (ε_{max} = 16.4%), and retractive stress at maximum strain (σ_{SF} at ε_{max} = 0.36 MPa) are displayed in Supplementary Table 2.

Junction density (v_j) is calculated by plugging in the stress from the static force at the strain peak ($\sigma_{SF\ at}\ \varepsilon_{max}$) measured by the instrument in the VLT into Supplementary Equation 1.

$$\sigma_{R_{max}} = \sigma_{SF\ at\ \varepsilon_{max}} = \nu_j RT\left(\alpha_{max} - \frac{1}{\alpha_{max}{}^2}\right) \tag{1}$$

Where: R is the universal gas constant, T is temperature, and α_{max} is the maximum extension ratio ($\alpha_{max} = L_{max}/L_o = \varepsilon_{max} + 1$). The entropy change upon stretching can be calculated by plugging α_{max} into Equation 1 of the main document. In order to determine the associated stored and released entropic energy density values (ΔS_S and ΔS_R, respectively) it is assumed that $\Delta H \approx 0$, thus the free energy ΔG per volume (J/m^3) will be $\Delta G = -T\Delta S$. Starting from the initial state before elongation, where $\Delta G \approx 0$ at temperature T_i (shown on Supplementary Fig. 2), then as temperature increases to $T\varepsilon_{max}$, strain reaches a maximum and the stored entropic energy density can be expressed as:

$$\Delta S_S = -T_{\varepsilon_{max}} S_{\varepsilon_{max}} + T_i S_i \tag{2}$$

Where: the S values (per volume) at each condition are determined from Equation 1 of the main document. When temperature increases to $T\varepsilon_{min}$ (Supplementary Fig. 2), retractive forces result in contraction to a local minimum ε_{min}, and the energy released can be determined from:

$$\Delta S_R = -T_{\varepsilon_{min}} S_{\varepsilon_{min}} + T_{\varepsilon_{max}} S_{\varepsilon_{max}} \tag{3}$$

Contraction and recovery percentages (in Supplementary Table 2) can be calculated by:

$$Contraction\% = \left(\frac{L_{max} - L_{min}}{L_i}\right) \times 100 \tag{4}$$

$$Recovery\% = \left(\frac{L_{max} - L_{min}}{L_{max} - L_i}\right) \times 100 \tag{5}$$

Optical Imaging

Supplementary Fig. 3 illustrates optical images of self-healing behavior (at 25 ^{0}C, ~50% relative humidity) as a function molecular weight function for (A) $M_W \approx$ 180 kDa, (B) $M_W \approx$ 72 kDa, (C) $M_W \approx$ 45 kDa, (D) $M_W \approx$ 32 kDa, and $M_W \approx$ 22 kDa TPU films. Wound closure of 72 kDa TPU films (Supplementary Fig. 3B) occurs within 40 minutes, consistent with the behavior of TPU fibers under the same conditions (Fig. 1A). Slower wound closure for 180 kDa TPU films (~2 hours) is a result of a slightly higher T_g, indicated by the slightly higher temperature for the VLT ε_{max} (Fig. 1D). For 45, 32, and 22 kDa TPU films (Supplementary Fig. 3 C-E), full wound closure does not

occur under these conditions. This is a result of poor shape recovery properties illustrated by the VLT from DMA (Fig. 1D, Supplementary Table 2). The damage initially narrows somewhat from limited recovery of entropic energy before subsequently widening in 32 kDa films (Supplementary Fig. 3C) and 45 kDa films (Supplementary Fig. 3B) and fibers (Fig. 1B). In 22 kDa TPU, the wound widens and shallows substantially over 24 hours. The widening of the wounds in low molecular weight TPUs is a result of flow driven by surface tension/energy[2-4] which acts to reduce surface area by decreasing the curvature at the bottom of a cut (Fig 4.). This effect from surface tension is more prominent in lower molecular weights due to lower viscosity, illustrated by Fig. 3 C and D.

The temperature and relative humidity dependence of self-repair in 72 kDa TPU is shown in Supplementary Fig. 4, at (A) 25 ^{0}C in air (~50% RH), (B) 21 ^{0}C in air (~50% RH), (C) 21 ^{0}C in dry atmosphere (~0-10% RH), (D) 11 ^{0}C in air (~40% RH), and (E) -11 ^{0}C in air. Self-repair in air (~50% RH) at 21 ^{0}C is slower than at 25 ^{0}C due to slower chain dynamics, thus slower viscoelastic recovery. Wound closure is substantially slowed in dry atmosphere at 21 ^{0}C, and full wound closure does not occur within 7 days. In general, the sensitivity of TPU to humidity is a result of not well understood so-called plasticization effect[5,6]. Experimentally, at ~50% RH TPU absorbs ~1.1 wt% H_20 (Supplementary Fig. 5B) and the T_g decreases by ~7 ^{0}C (Supplementary Fig. 5A) thus leading to the increase of molecular mobility. Wound closure is stalled at 11 ^{0}C and -11 ^{0}C because the conformational entropic forces are unable to overcome the interactions between chains in the glassy state. For the same reason, when a portion of PTHF is substituted by 1,4 butanediol (BDO), which increases T_g, the temperature required to initiate self-repair increases (Supplementary Figs. 9 and 10), attributed to increased chain rigidity/hard segment content. This type of behavior is more akin to reversible plasticity shape memory (RPSM)[7] assisted repair, but also note chemical modification to alter T_g allows tuning of the temperature range for viscoelastic shape memory (VESM) behavior for applications in non-ambient conditions.

Infrared Spectroscopy (IR)

2D-FTIR correlation spectra[8] were calculated using 2DShige software (2Dshige © Shigeaki Morita, Kwansei-Gakuin University, 2004-2005). Asynchronous 2D-FTIR correlation spectra were calculated using from the following equation (using no reference spectrum):

$$\Psi(\nu_1, \nu_2) = \frac{1}{\pi}[y_1(\nu_1)y_2(\nu_2) - y_2(\nu_1)y_1(\nu_2)] \quad (6)$$

where: ν_1 and ν_2 represent two wavenumbers, and y_1 and y_2 represent IR intensities in the first and second spectra, respectively [8]. To analyze spectroscopic changes upon damage, spectra of undamaged TPU were input as y_1, and spectra collected after perturbation (damage) were input as y_2. To analyze changes upon repair, spectra of damaged TPU were input as y_1, and spectra collected after self-repair were input as y_2. When the ratio of band intensities at ν_1 and ν_2 (cm^{-1}) changes going from y_1 to y_2, two cross-peaks will be observed. The presence of a positive cross-peak indicates the intensity ratio $y_2(\nu_2)/y_2(\nu_1) > y_1(\nu_2)/y_1(\nu_1)$ and a negative cross-peak means $y_2(\nu_2)/y_2(\nu_1) < y_1(\nu_2)/y_1(\nu_1)$.

Synchronous 2D-FTIR correlation spectra were calculated using the following equation (using the average spectrum as the reference):

$$\Phi(\nu_1, \nu_2) = [z_1(\nu_1)z_1(\nu_2) + z_2(\nu_1)z_2(\nu_2)] \quad (7)$$

Where: z_1 and z_2 represent the difference between IR intensities in the first and second spectra, respectively, and the average intensity (reference spectrum). The presence of positive cross-peak indicates ν_1 and ν_2 (cm^{-1}) change in the same direction (either both increase or both decrease) going from y_1 to y_2, while a negative cross-peak indicates that ν_1 and ν_2 change in opposite directions (one increases when the other decreases).

Supplementary Figs. 11-13 show attenuated total reflectance (ATR) FTIR spectra which illustrate spectral changes upon damage (A) and during repair (B) in the urethane amide (UA) I region (11), UA II region (12), and UA III region (13). All three regions show H-bonding UA bands (1682 cm^{-1}, 1540 cm^{-1}, and 1244 cm^{-1}) decrease upon damage while free UA bands (1719 cm^{-1}, 1505 cm^{-1}, and 1224 cm^{-1}) increase. During repair, H-bonding UA bands (1682 cm^{-1}, 1540 cm^{-1}, and 1244 cm^{-1}) increase while free UA bands (1719 cm^{-1}, 1505 cm^{-1}, and 1224 cm^{-1}) decrease. These results indicate H-bonding decreases upon damage of TPU, but recovers during self-repair, as shown by the 2D correlation spectra calculated from these results in Fig 3.

Supplementary Figs. 6 (A, C, D) and 7 (A, B) show IR spectra from internal reflection infrared imaging (IRIRI) immediately following damage as a function of distance from the damage center. In these spectra, it is seen that at the center of the wound, all band positions are all shifted about 1-2 cm^{-1} to higher wavenumbers compared to undamaged TPU or near the edge of damage. However, underneath these shifts, changes in H-bonding can be observed, as illustrated by Supplementary Figs. 14 and 15 which shows peak fitting results in the urethane amide II region. Both the H-bonding and non H-bonding band positions shift to higher wavenumber inside the damaged area, but the intensity of the H-bonding band is decreased while the non H-bonding is increased (Supplementary Fig. 15). Thus, these results are consistent with H-bonding changes seen in Fig. 3 upon damage. However, the shifts of band positions prevents detection of H-bonding changes by 2D correlation IR spectra from IRIRI results, shown in Supplementary Fig. 16. Unlike the synchronous data in Fig. 3, A1-A3, the synchronous spectra in Supplementary Fig. 16 A-C shows that the high-wavenumber side of each band are positively correlated with each other, and inversely correlated with the low-wavenumber side of each band. The asynchronous spectra in Supplementary Fig. 16, D-F show that upon damage, the high-wavenumber side of each band increases relative to the low wave number side. This characteristic “butterfly” pattern[9] is a result of the 1-2 cm^{-1} shift to higher wavenumber upon damage in each band seen in the IRIRI 1-D spectra, which overshadows the changes in hydrogen bonding in the 2D correlation spectra. Also, some additional peaks from splitting are observed due to the band position shift in the asynchronous data.

Supplementary Tables

Supplementary Table 1. Tensile analysis results: Modulus (J), tenacity (σ-failure), and failure strain (ε-failure) from tensile analysis for undamaged and self-healed TPU fibers of 72 and 45 kDa.

		TPU fibers $M_W \approx 72$ kDa	Tenacity		TPU fibers $M_W \approx 45$ kDa	Tenacity	
		J (Modulus) (mN/tex)	σ-failure (mN/tex)	ε-failure (%)	J (Modulus) (mN/tex)	σ-failure (mN/tex)	ε-failure (%)
UNDAMAGED	AVERAGE	299	21.0	279	267	11.90	200
	STD DEV	+/- 70	+/- 4.06	+/- 39	+/- 110	+/- 2.36	+/- 40
HEALED	AVERAGE	302	21.3	288	237	7.55	97
	STD DEV	+/- 50	+/- 1.9	+/- 23	+/- 119	+/- 2.62	+/- 76

*TPU density at ambient conditions = 1.09 g/cm

Supplementary Table 2. Summary of TPU VLT properties obtained from DMA as a function of molecular weight.

M_W (kDa)	v_j* (mol/m^3)	tan δ_{max}	ε_{max} (%)	ε_{min} (%)	Contraction (%)	Recovery (%)	$\sigma_{SF\ at\ \varepsilon max}$ (MPa)	ΔS_S (kJ/m^3)	ΔS_R (kJ/m^3)
22	78.4**	1.95	29.5	25.1	4.4	15.0	0.143	22.7**	5.4
32	153.5**	1.87	24.4	16.5	7.8	32.4	0.240	31.2**	15.6
45	199.0**	1.89	19.8	11.8	8.0	40.6	0.261	27.3**	16.7
72	321.1	1.79	16.4	5.6	10.8	66.9	0.358	30.7	26.7
180	456.3	1.75	16.1	4.3	11.8	73.9	0.505	42.4	39.0

*v_j calculated from $\sigma_{R_{max}} = \sigma_{SF\ at\ \varepsilon_{max}} = \nu_j RT\left(\alpha - \frac{1}{\alpha^2}\right)$

**Because the rubber elasticity equations used to calculate v_j and ΔS_S assume all elongation (α) results in conformational changes and thus stored entropic energy, these values are subject to considerable error for low molecular weights due to observed non-recovered deformation resulting from chain slippage/flow. The significant disparities between calculated ΔS_S and ΔS_R values for low molecular weights also show that this is the case. This is also why 22, 32, and 45 kDa TPU are unable to fall properly on the shape memory prediction plane in Fig. 1E.

Supplementary Figures

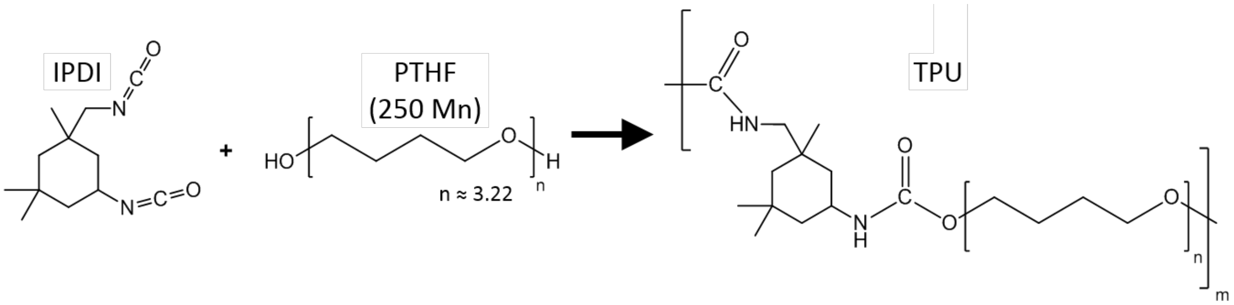

Supplementary Fig. 1. Synthesis of TPU from isophorone diisocyanate (IPDI) and polytetrahydrofuran (PTHF) M_n = 250 Da.

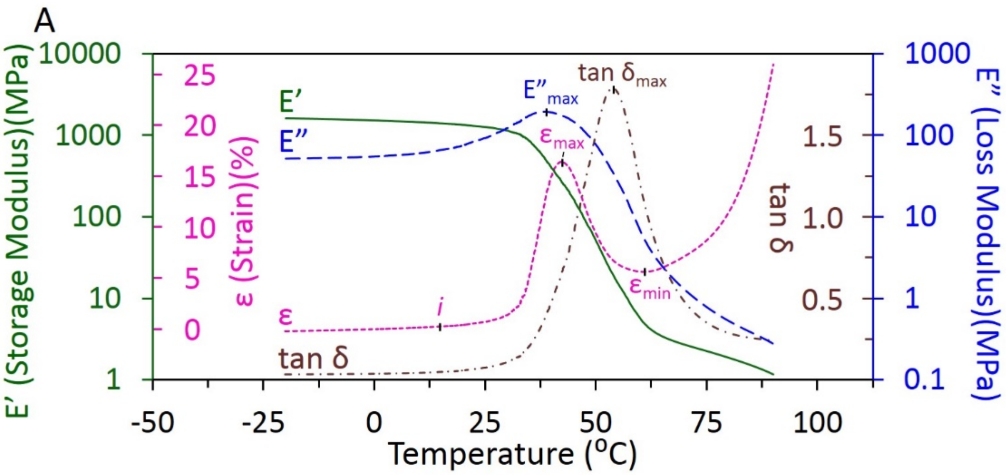

B	M_W (kDa)	v_j (mol/m³)	tan δ_{max}	ε_{max} (%)	σ_{SF} at ε_{max} (MPa)	ΔS_S (kJ/m³)	ΔS_R (kJ/m³)	$T_{E''max}$ (°C)	$T_{\varepsilon max}$ (°C)	$T_{tan\ \delta max}$ (°C)
TPU	72	321.1	1.79	16.4	0.358	30.7	26.7	38.5	42.3	53.8

Supplementary Fig. 2. (A) Tensile strain-controlled dynamic mechanical analysis (DMA) storage modulus (E'), loss modulus (E"), tan δ, and sample length changes (ε) of 72 kDa TPU film, showing DMA viscoelastic length transition (VLT) behavior. (B) TPU junction density (v_j), tan δ_{max}, as well as VLT stored strain (ε_{max}), stress (σ_{SF} at ε_{max}), stored (ΔS_S) and released (ΔS_R) entropic energy density, and the temperatures of maximum loss modulus (TE''_{max}), maximum strain ($T\varepsilon_{max}$), and tan δ_{max} (Ttan δ_{max}).

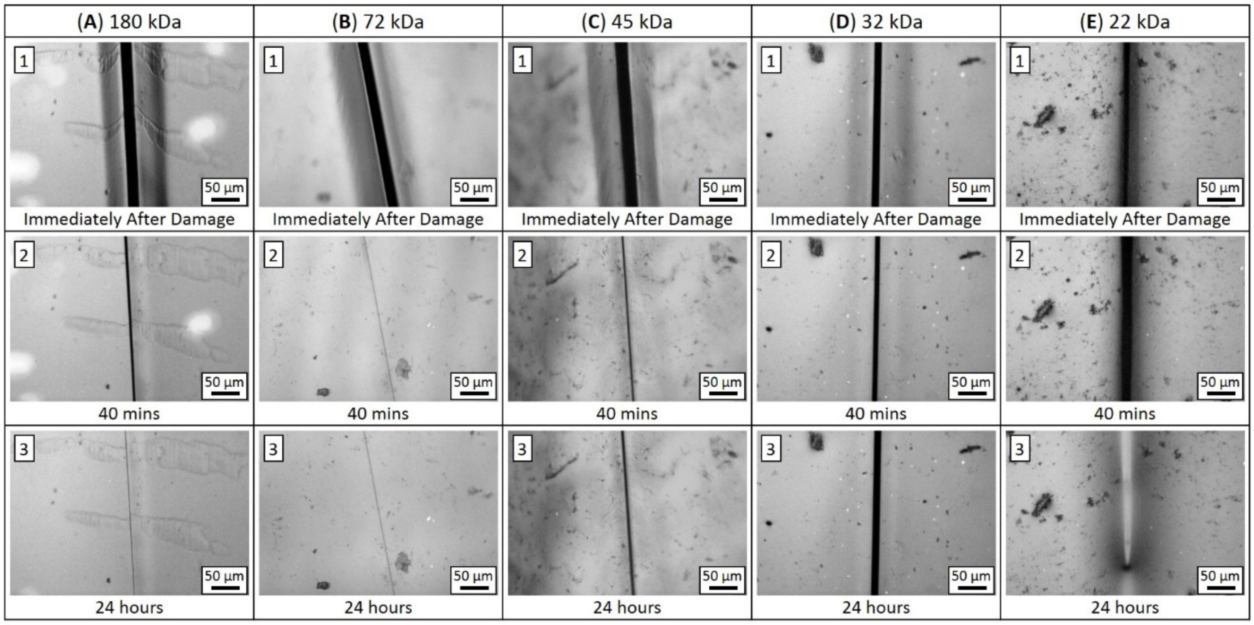

Supplementary Fig. 3. Optical images of TPU films. Molecular weight dependence of self-healing: (A) $M_W \approx 180$ kDa; (B) $M_W \approx 72$ kDa; (C) $M_W \approx 45$ kDa; (D) $M_W \approx 32$ kDa; $M_W \approx 22$ kDa. 1→ immediately after damage; 2→ 40 minutes after damage; 3→ 24 hours after (at 25 ^{0}C, ~50% relative humidity).

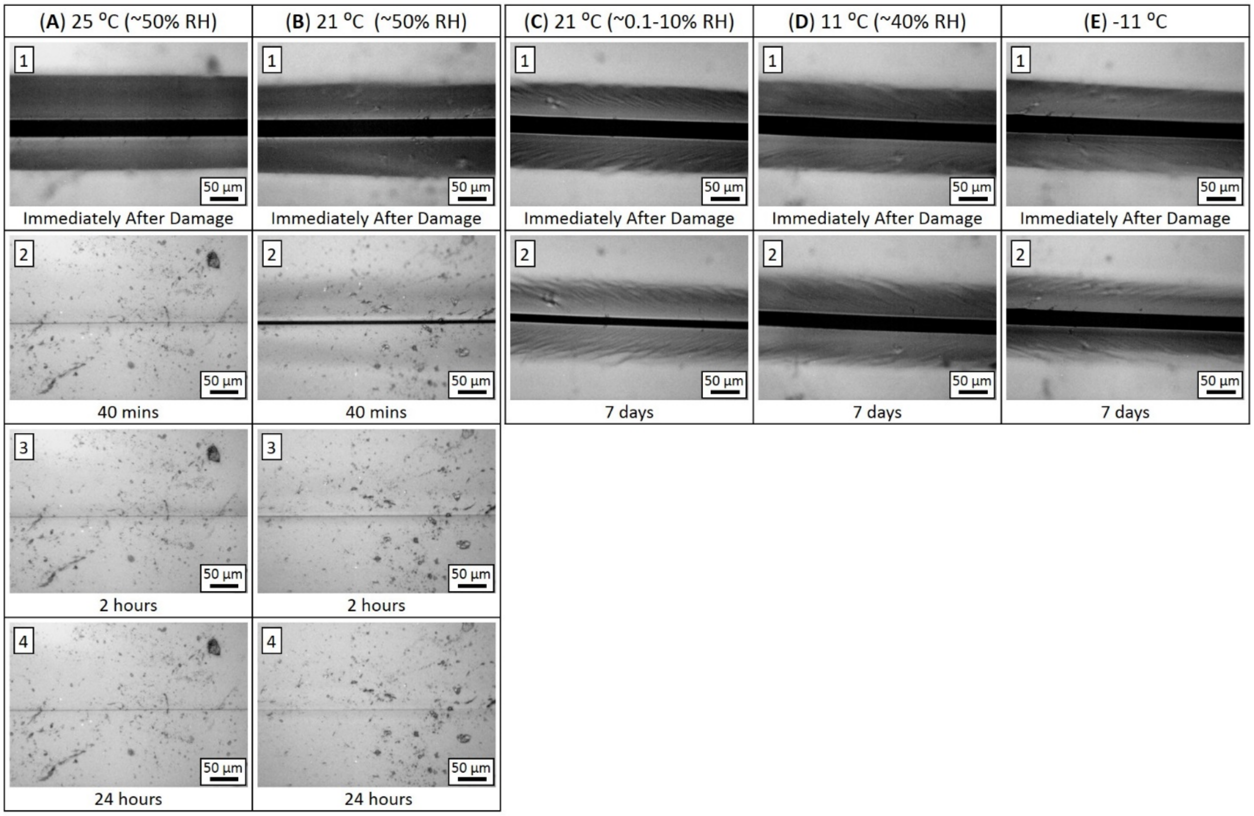

Supplementary Fig. 4. Optical images of TPU films ($M_W \approx 72$ kDa). Temperature and relative humidity (RH) dependence of self-healing: (A) 25 ^{0}C in air (~50% RH); (B) 21 ^{0}C in air (~50% RH); (C) 21 ^{0}C in dry atmosphere (~0-10% RH); (D) 11 ^{0}C in air (~40% RH); (E) -11 ^{0}C in air.

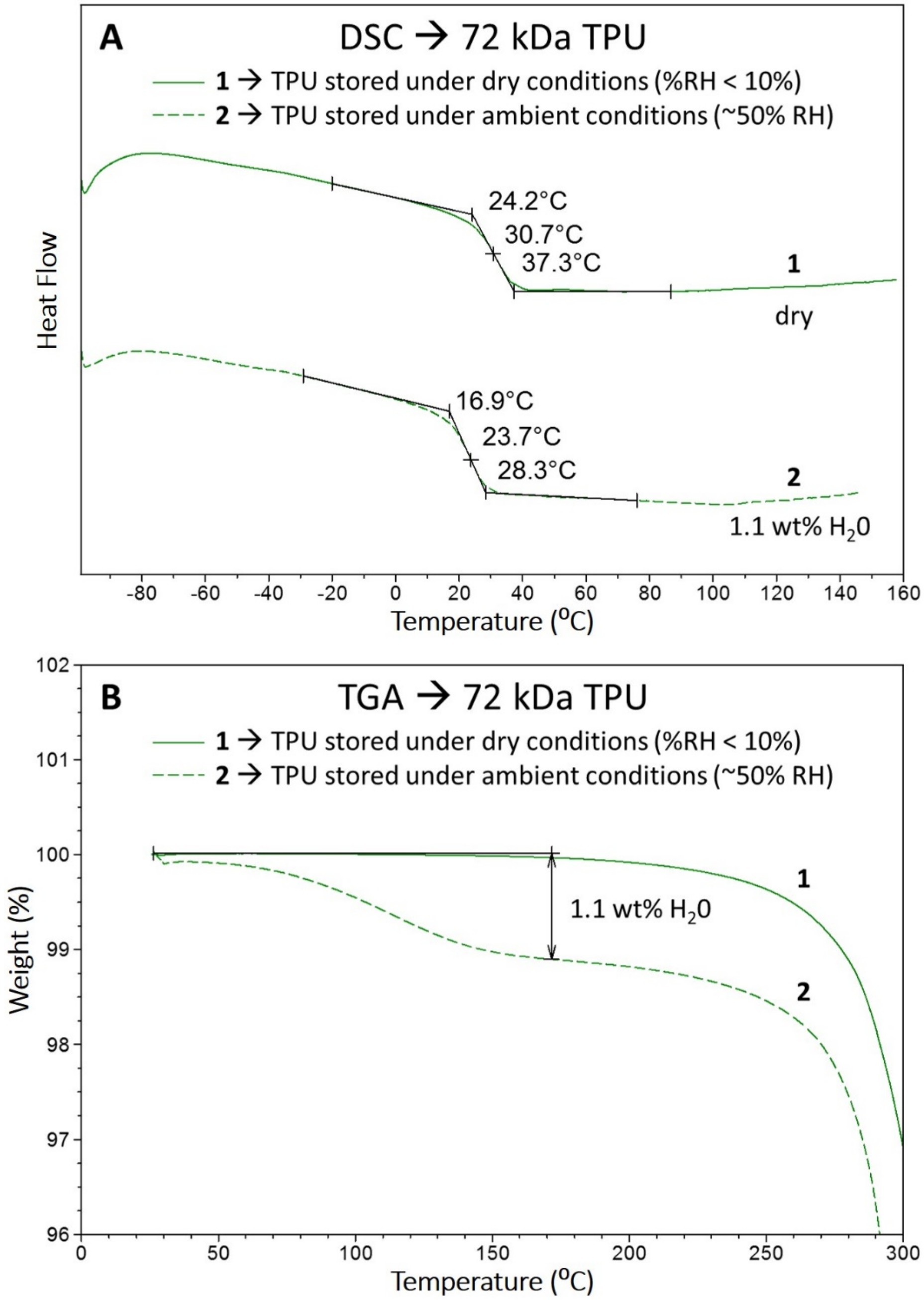

Supplementary Fig. 5. (A) DSC and (B) TGA results of M_W ≈ 72 kDa TPU, (1) after storing under ambient conditions (~50% RH), and (2) after storing under dry conditions (%RH < 10%)

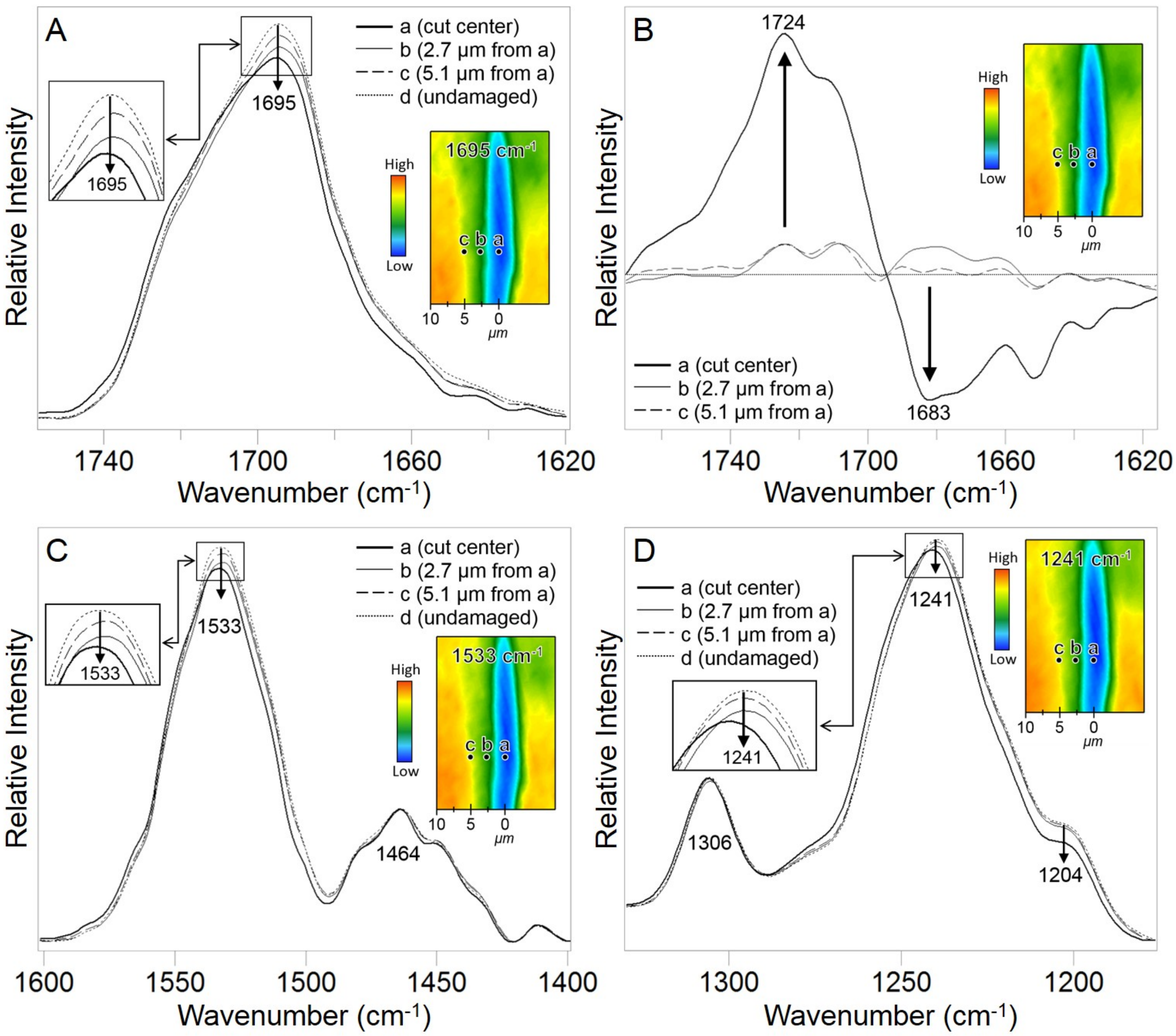

Supplementary Fig. 6. IR image of cut cross-section and (A) 1695 cm^{-1}, (C) 1533 cm^{-1}, and (D) 1241 cm^{-1} band changes recorded as a function of distance from the center of the damage from IRIRI. (B) The intensity changes of the 1722 cm^{-1} and 1683 cm^{-1} bands as a function of the distance from the center of cut (relative to 1695 cm^{-1}) from IRIRI.

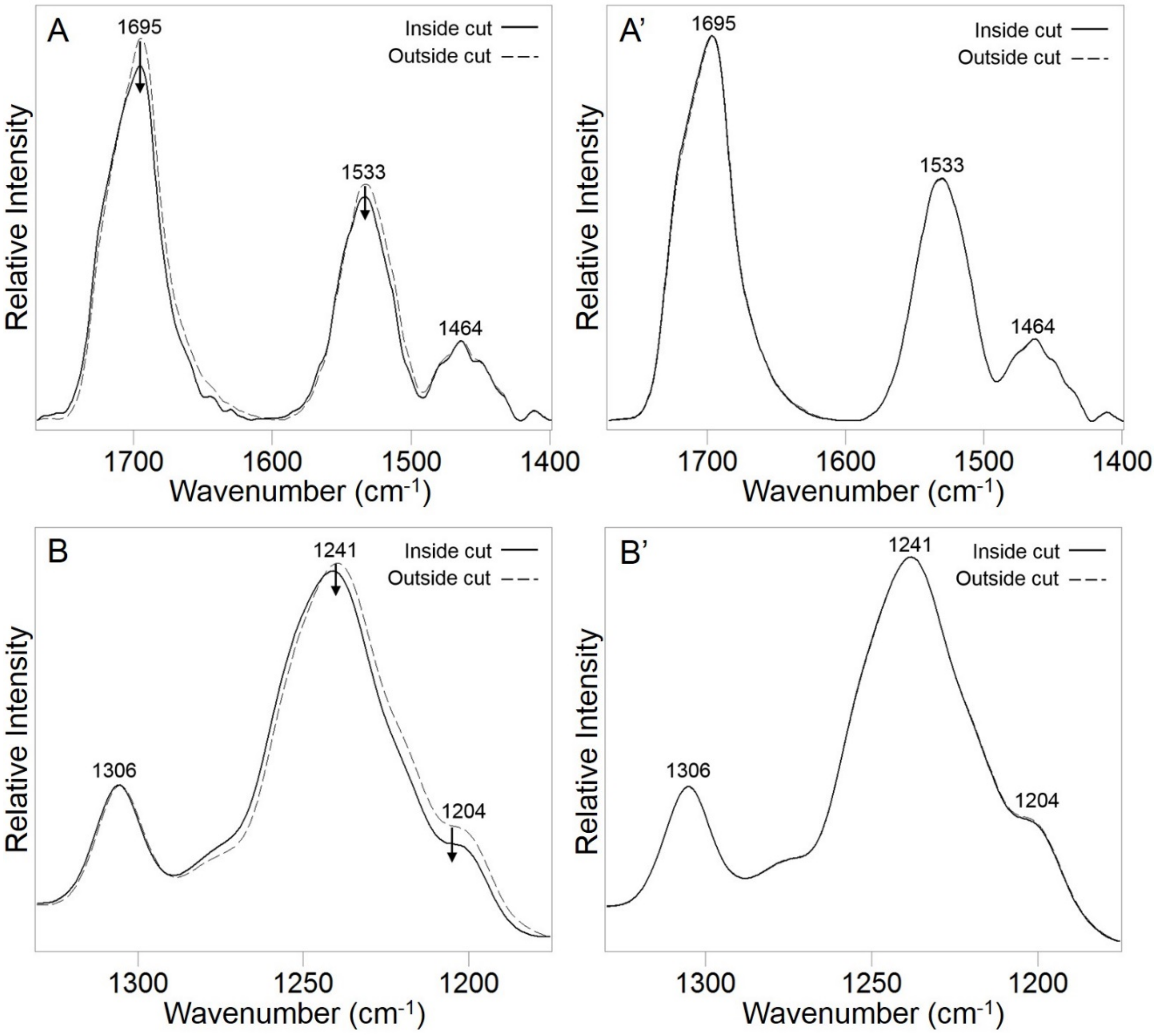

Supplementary Fig. 7. IR spectra collected inside the scratch (solid line) and outside the scratch in the surrounding area (dashed line) immediately after damage in the 1760-1400 cm^{-1} region (A) and 1340-1170 cm^{-1} region (B), and in the same regions after recovery of damage (A', B').

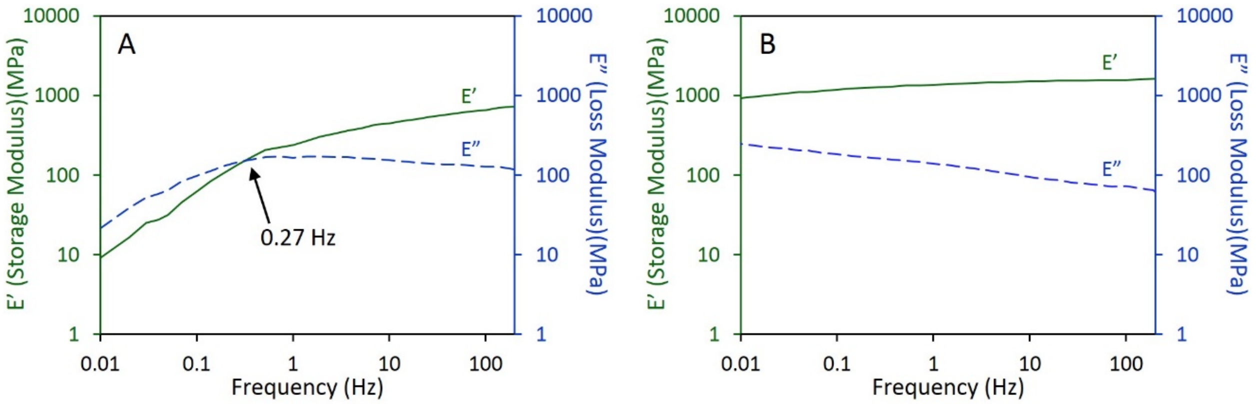

Supplementary Fig. 8. Isothermal frequency sweep experiments for 72 kDa TPU from 200 to 0.01 Hz, at (A) 25 ^{0}C and ~50 %RH (A), and (B) 21 ^{0}C in dry atmosphere (%RH < 10%).

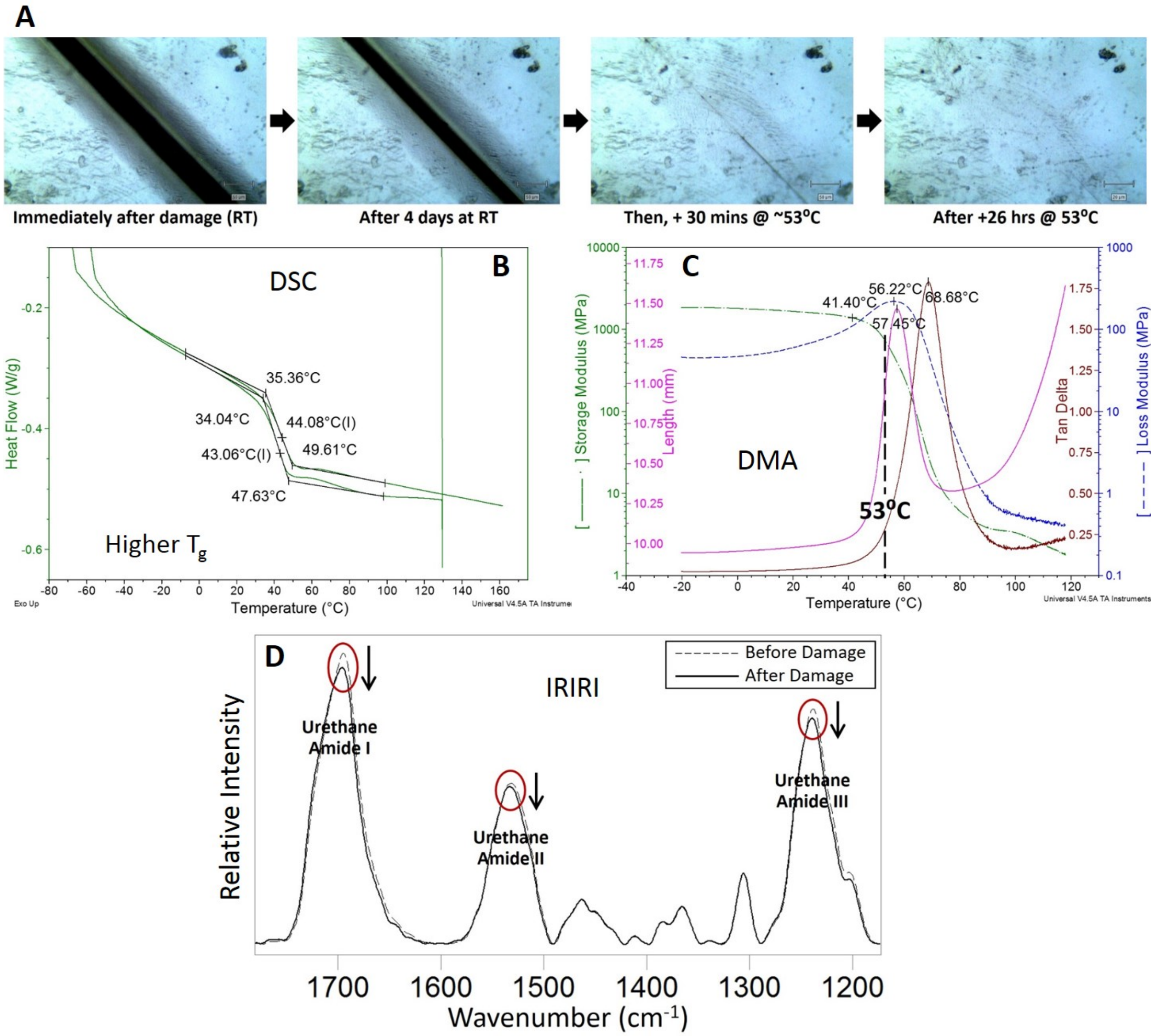

Supplementary Fig. 9. (A) Self-healing (initially at room temperature (RT) for 4 days, followed by 26 hours at 53 ^{0}C), (B) DSC, (C) DMA, and (D) IRIRI results for TPU (20 mol% of PTHF substituted for by 1,4 butanediol).

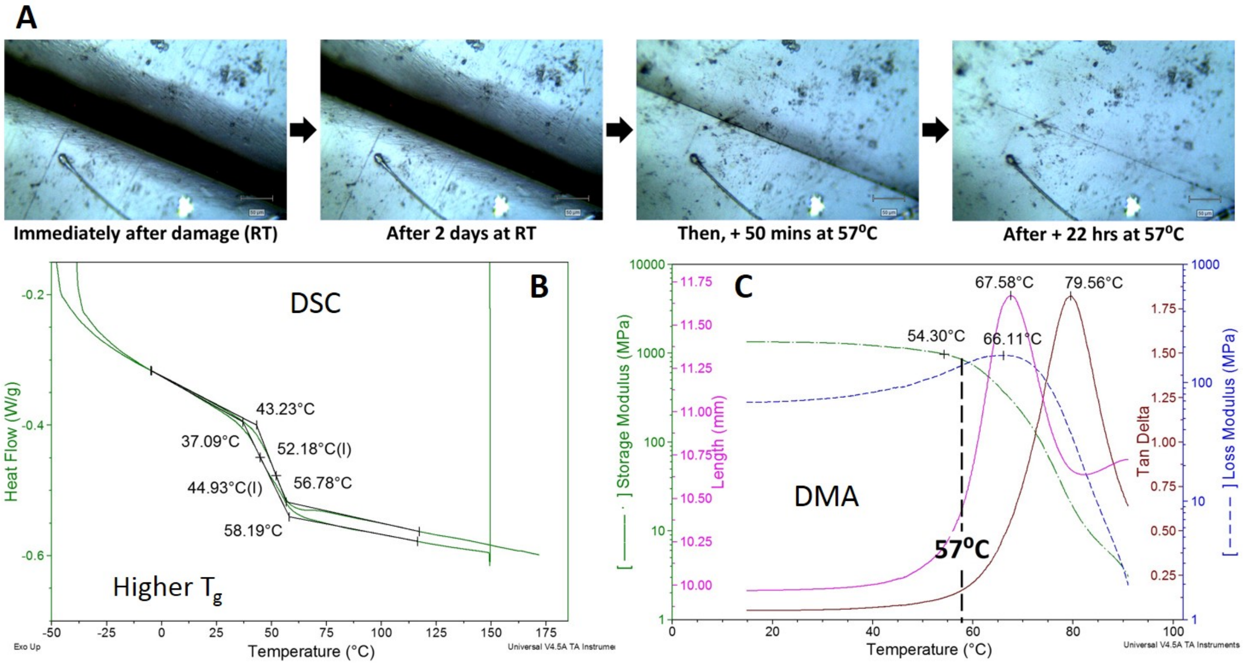

Supplementary Fig. 10. (A) Self-healing (initially at room temperature (RT) for 2 days, followed by 22 hours at 57 ^{0}C), (B) DSC, and (C) DMA for TPU (35 mol% of PTHF substituted for by 1,4 butanediol).

Supplementary Table 3: Relevant IR and Raman vibrational bands assignments detected in TPU.

IR	Raman	Tentative Band Assignments
Wavenumber (cm^{-1})		
3321		Stretch N-H + stretch O-H
2945		Stretch C-H (asym)
2857		Stretch C-H (sym)
1722		Stretch asymmetric C=O (free urethane amide I)
1685		Stretch asymmetric C=O (H-bonding urethane amide I)
1538		Stretch C-N + δ N-H (H-bonding urethane amide II)
1510		Stretch C-N + δ N-H (free urethane amide II)
1462		δ asymmetric C-H (CH_2 scissor)
1449		δ asymmetric C-H (CH_3 bending def)
1305	1298	(N-H def?)
1244		Stretch asym N-CO-O + Stretch (C-O-C) (H-bonding urethane amide III)
1224		Stretch asym N-CO-O + Stretch (C-O-C) (free urethane amide III)
1204	1204	(N-H def?)
1110		Stretch C-O-C (PTHF)

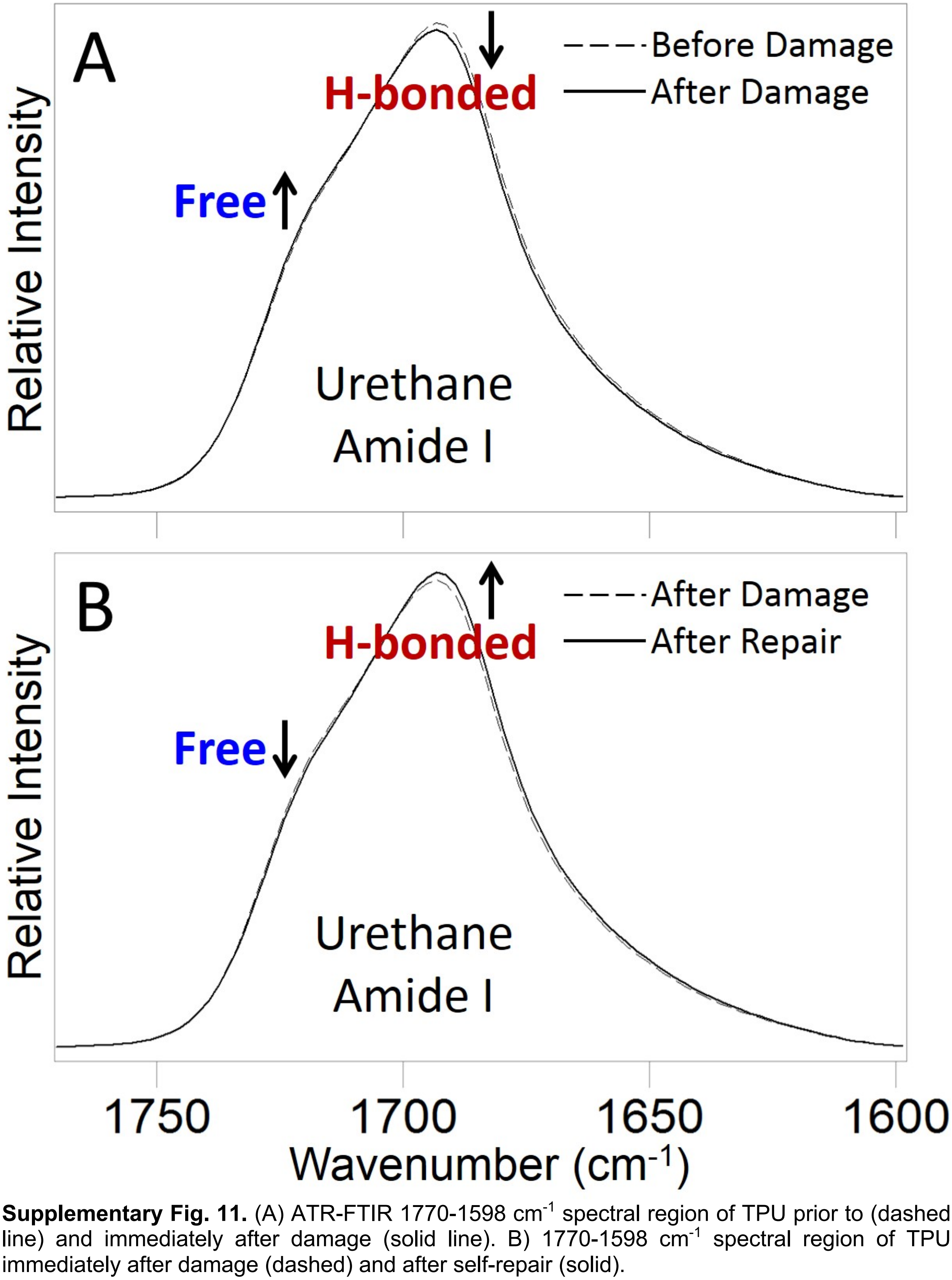

Supplementary Fig. 11. (A) ATR-FTIR 1770-1598 cm^{-1} spectral region of TPU prior to (dashed line) and immediately after damage (solid line). B) 1770-1598 cm^{-1} spectral region of TPU immediately after damage (dashed) and after self-repair (solid).

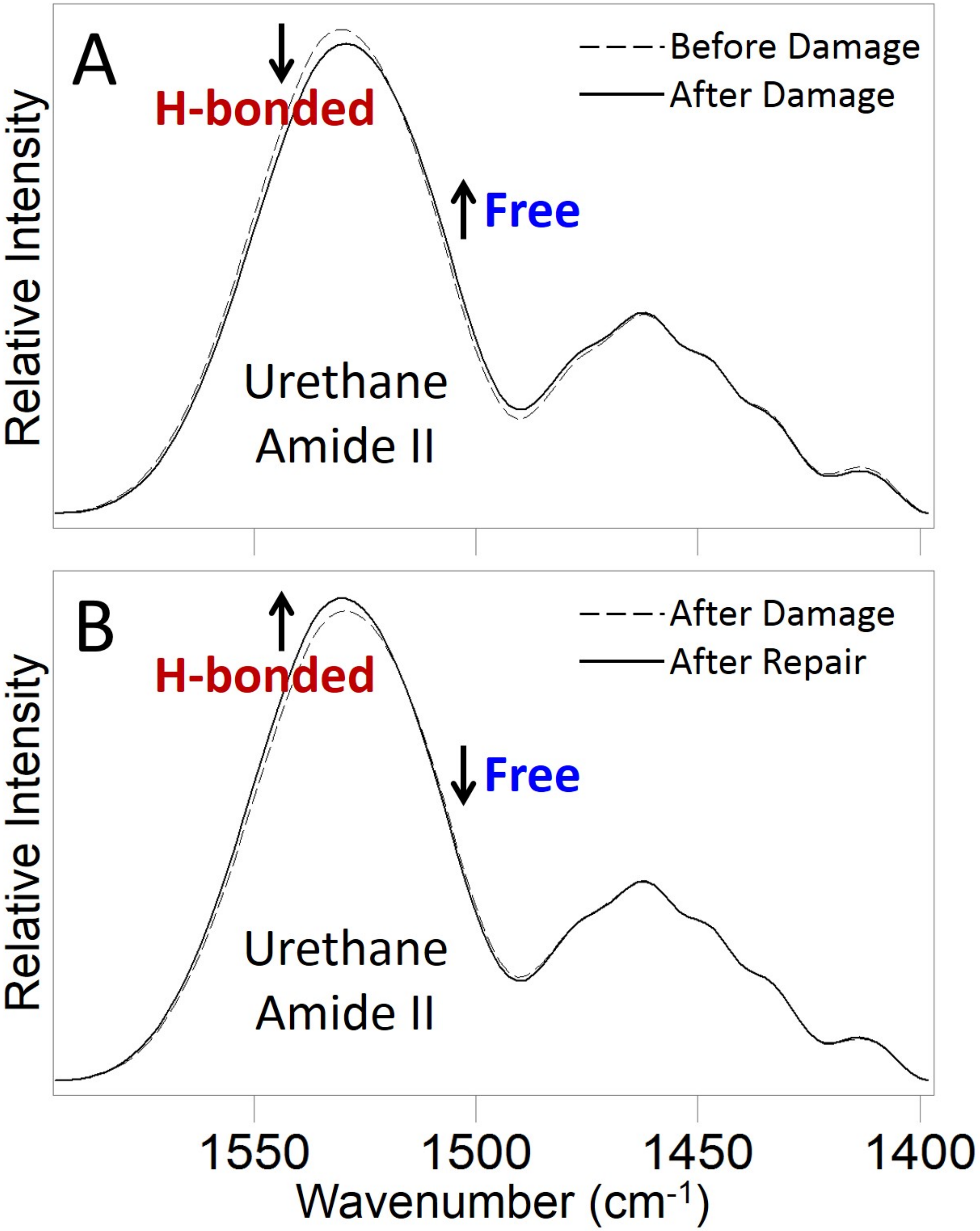

Supplementary Fig. 12. (A) ATR-FTIR 1595-1397 cm^{-1} spectral region of TPU prior to (dashed line) and immediately after damage (solid line). (B) 1595-1397 cm^{-1} IR spectral region of TPU immediately after damage (dashed) and after self-repair (solid).

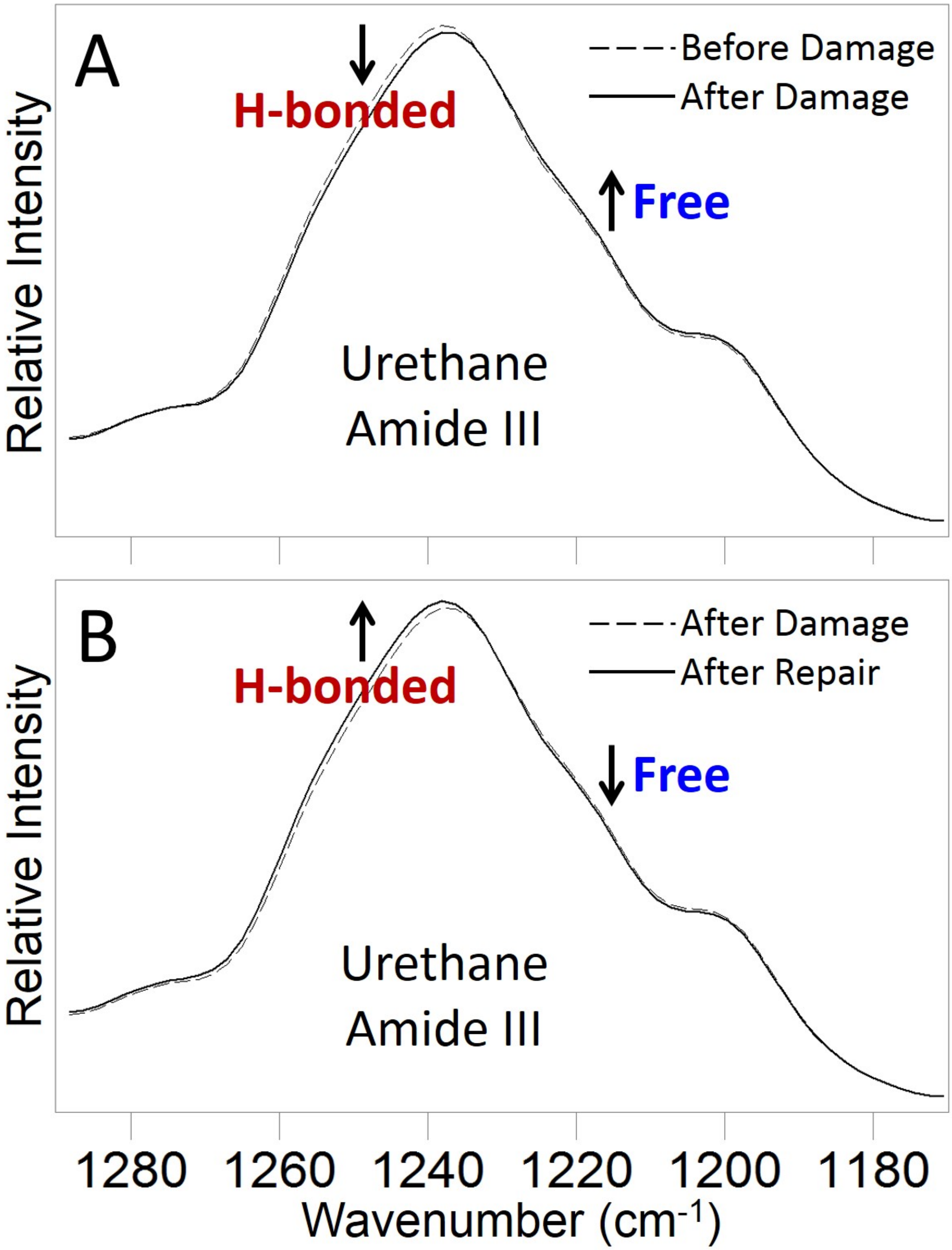

Supplementary Fig. 13 (A) ATR-FTIR 1290-1170 cm^{-1} spectral region of TPU prior to (dashed line) and immediately after damage (solid line). (B) 1290-1170 cm^{-1} IR spectral region of TPU immediately after damage (dashed) and after self-repair (solid).

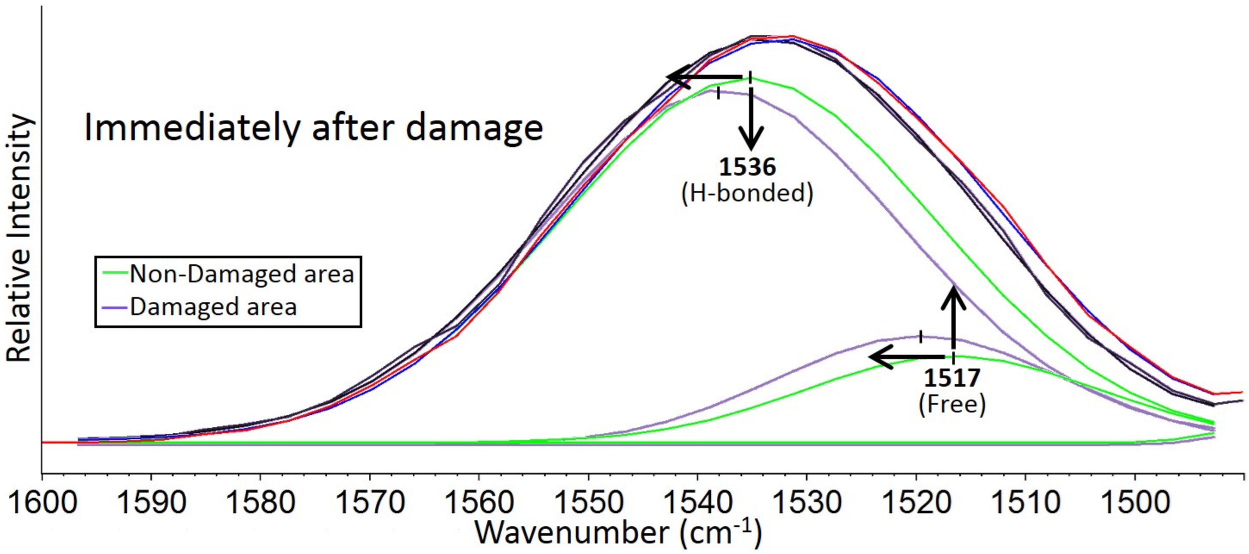

(+/-) = Damaged - Undamaged

Band	Centre (+/-)	Width (+/-)	Relative Height (+/-)		Relative Area (+/-)
1536 (**H-B**)	**+2.383**	-1.215	**-0.0202**		**-0.7958**
1517 (**F**)	**+2.928**	-0.777	**+0.0119**		**+0.3304**
$\left(\frac{H - Bonded}{Free}\right) \rightarrow$ 1536/1517			**-0.964**		**-1.054**
			By Height		By Area
		H-Bonded%	**-4.30%**		**-3.03%**
		Free%	**+4.30%**		**+3.03%**

-**Non-damaged** area has **more H-bonded** groups

-**Damaged** area has **more free** groups and bands are at **higher** vibrational **frequencies**

Supplementary Fig. 14. Peak fitting results of damaged and non-damaged TPU of 1530 cm^{-1} band (urethane amide II) in IRIRI.

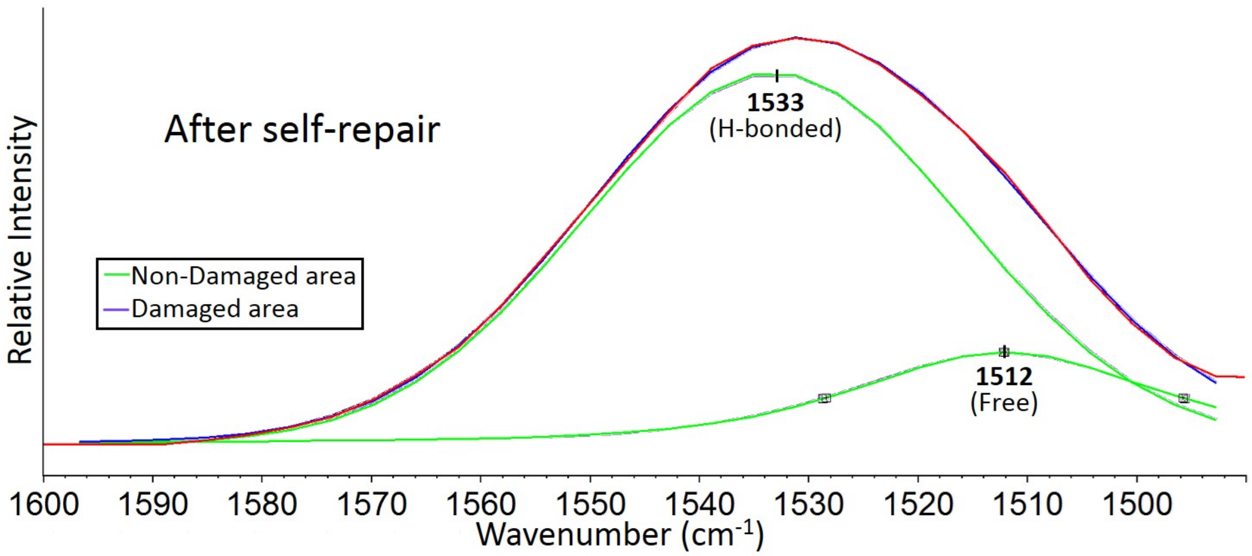

(+/-) = Damaged - Undamaged

Band	Centre (+/-)	Width (+/-)	Relative Height (+/-)		Relative Area (+/-)
1533 (**H-B**)	**-0.0063**	+0.187	**-0.0007**		**+0.0212**
1512 (**F**)	**+0.1100**	+0.404	**-0.0004**		**-0.0412**
1533/1512			**+0.0165**		**+0.0642**

	By Height		By Area
H-Bonded%	**+0.07%**		**+0.26%**
Free%	**-0.07%**		**-0.26%**

$\left(\frac{H - Bonded}{Free}\right)$ → 1533/1512

These differences are negligible

Supplementary Fig. 15. Peak fitting results of healed and non-damaged TPU of 1530 cm^{-1} band (urethane amide II) from IRIRI.

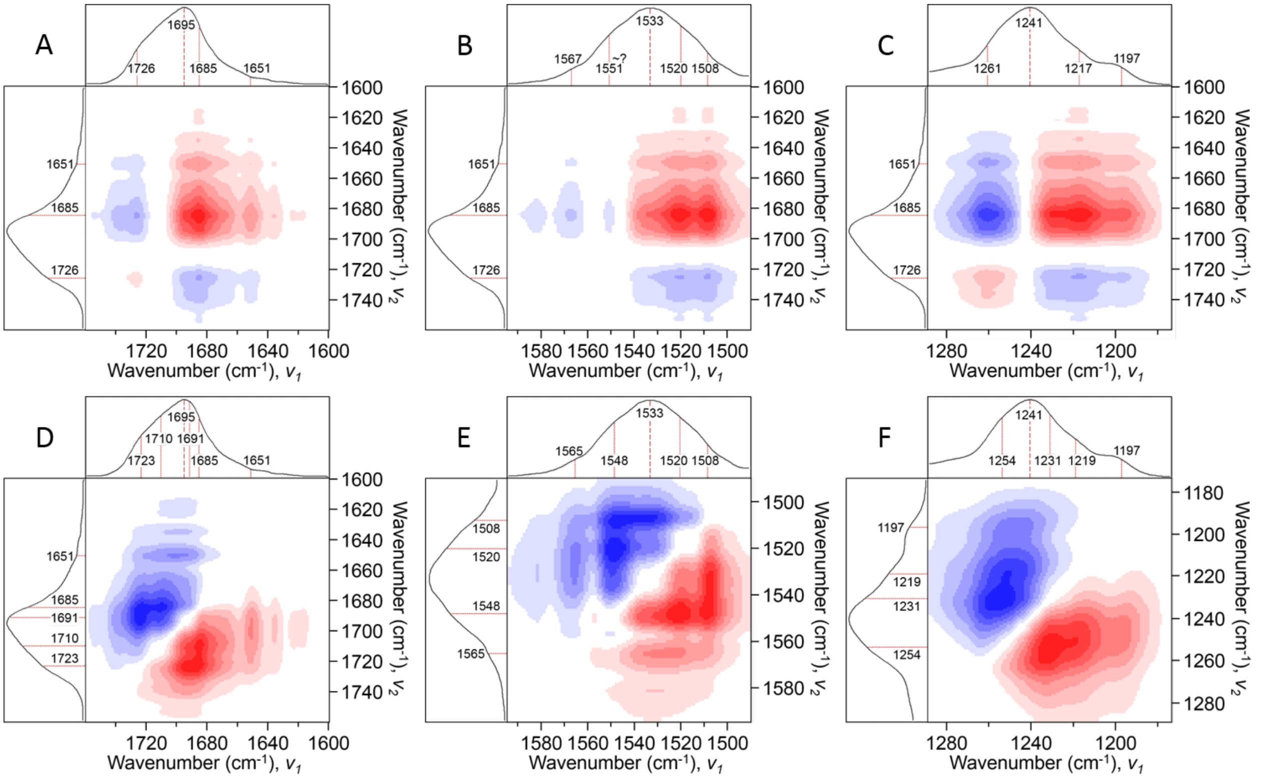

Supplementary Fig. 16. 2D-FTIR correlation spectra for TPU (IRIRI-FTIR). (A, B, and C) are synchronous spectra (using the average spectrum as the reference spectrum) of (A) 1760-1600 cm^{-1} (urethane amide I) vs. 1720-1600 cm^{-1} (urethane amide I), (B) 1595-1490 cm^{-1} (II) vs. 1720-1600 cm^{-1} (I), (C) 1329-1173 cm^{-1} (III) vs. 1760-1600 cm^{-1}. (D, E, F) are asynchronous spectra (using no reference spectrum) of (D) 1760-1600 cm^{-1} (I), (E) 1595-1490 cm^{-1} (II), (F) 1329-1173 cm^{-1} (III).

Supplementary References

1 Hornat, C. C., Yang, Y. & Urban, M. W. Quantitative Predictions of Shape-Memory Effects in Polymers. *Advanced Materials* **29**, 1603334 (2017).

2 Fakhraai, Z. & Forrest, J. A. Measuring the surface dynamics of glassy polymers. *Science* **319**, 600-604 (2008).

3 Chai, Y. *et al.* A direct quantitative measure of surface mobility in a glassy polymer. *Science* **343**, 994-999 (2014).

4 Paretkar, D., Xu, X., Hui, C.-Y. & Jagota, A. Flattening of a patterned compliant solid by surface stress. *Soft matter* **10**, 4084-4090 (2014).

5 Huang, W. M., Yang, B., An, L., Li, C. & Chan, Y. S. Water-driven programmable polyurethane shape memory polymer: demonstration and mechanism. *Applied Physics Letters* **86**, 114105 (2005).

6 Yang, B., Huang, W. M., Li, C. & Li, L. Effects of moisture on the thermomechanical properties of a polyurethane shape memory polymer. *Polymer* **47**, 1348-1356 (2006).

7 Rodriguez, E. D., Luo, X. & Mather, P. T. Linear/network poly (ε-caprolactone) blends exhibiting shape memory assisted self-healing (SMASH). *ACS applied materials & interfaces* **3**, 152-161 (2011).

8 Noda, I. Generalized two-dimensional correlation method applicable to infrared, Raman, and other types of spectroscopy. *Applied spectroscopy* **47**, 1329-1336 (1993).

9 Noda, I. Progress in two-dimensional (2D) correlation spectroscopy. *Journal of Molecular Structure* **799**, 2-15 (2006).
